# Abnormal gas-liquid-solid phase transition behaviour of water observed with *in situ* environmental SEM

**DOI:** 10.1038/srep46680

**Published:** 2017-04-24

**Authors:** Xin Chen, Jiapei Shu, Qing Chen

**Affiliations:** 1 Key Laboratory for Ultrafine Materials of Ministry of Education and Shanghai Key Laboratory of Advanced Polymeric Materials, School of Materials Science and Engineering, East China University of Science and Technology, Shanghai 200237, China; 2 State Key Laboratory of Functional Materials for Informatics, Shanghai Institute of Microsystem and Information Technology, Chinese Academy of Sciences, 865 Changning Road, Shanghai 200050, China; 3 Key Laboratory for the Physics and Chemistry of Nanodevices and Department of Electronics, Peking University, Beijing 100871, China; 4 Academy for Advanced Interdisciplinary Studies, Peking University, Beijing 100871, China

## Abstract

Gas-liquid-solid phase transition behaviour of water is studied with environmental scanning electron microscopy for the first time. Abnormal phenomena are observed. At a fixed pressure of 450 Pa, with the temperature set to −7 °C, direct desublimation happens, and ice grows continuously along the substrate surface. At 550 Pa, although ice is the stable phase according to the phase diagram, metastable liquid droplets first nucleate and grow to ~100–200 μm sizes. Ice crystals nucleate within the large sized droplets, grow up and fill up the droplets. Later, the ice crystals grow continuously through desublimation. At 600 Pa, the metastable liquid grows quickly, with some ice nuclei floating in it, and the liquid-solid coexistence state exists for a long time. By lowering the vapour pressure and/or increasing the substrate temperature, ice sublimates into vapour phase, and especially, the remaining ice forms a porous structure due to preferential sublimation in the concave regions, which can be explained with surface tension effect. Interestingly, although it should be forbidden for ice to transform into liquid phase when the temperature is well below 0 °C, liquid like droplets form during the ice sublimation process, which is attributed to the surface tension effect and the quasiliquid layers.

Water has attracted extensive research interests over the years due to its critical role in a broad range of scientific disciplines and in supporting life on earth^1^,^2^,^3^,^4^,^5^,^6^. Besides, the water system is also important due to the rich stable/metastable phase behaviour^1^, which brings about opportunities to find out new thermodynamic properties and new phase transformation mechanisms, with many puzzles still to be explored^2^,^6^. Although the condensation and vapourization of water is a pretty old topic, it is still worth studying with new methods^7^,^8^. Understanding the water phase transition behaviour highly relies on imaging techniques^9^,^10^. As optical microscopes have limited image resolution and depth of field, *in situ* transmission electron microscope (TEM) method has been developed to study the evolution of ice crystals during cooling procedures^8^. However, due to electron scattering effect, only very thin sample can be observed by *in situ* TEM, while, samples with nanoscale thickness may have different properties from the bulk counterparts. Furthermore, performing low temperature *in situ* TEM experiment with a variable pressure is technically challenging, and so far no such experiment on water/ice system has been reported.

Scanning electron microscope (SEM), with a large sample chamber, can be used to study thick samples. Environmental SEM (ESEM) allows some low pressure water vapour to be introduced into the vacuum chamber, thus is very useful for studying water-containing systems. The technology has now been widely used in biological research^11^,^12^, food study^13^, and material synthesis^14^. ESEM has been used to observe ice surface structures^15^, and ice growth behaviour^16^,^17^. Surface ablation of ice crystal facets have also been observed below −30 °C^16^. However, most previous studies on ice with ESEM have been performed below 0.1 Pa and at temperatures much lower than 0 °C^15^, which only allow *in situ* observation of vapour-liquid-solid transitions in low melting temperature systems (e.g. the partially molten sulfuric system)^18^, but are unable to study the vapour-liquid-ice system of pure water. Recently, the instrumental developments allow the exploration of pressures higher than 700 Pa, at temperatures around 0 °C, which covers the triple point of water^15^. With the instrumental advancement, water nanocondensation on flat and patterned surfaces has been observed^19^,^20^. While, the observation of gas-liquid-solid phase transitions of water with ESEM has not been reported in the literature, and is still highly desired.

In this paper, we report the first *in situ* ESEM study on the gas-liquid-solid phase transitions of water. This is achieved by decreasing the sample temperature with a cold stage, and controlling the vapour pressure in the ESEM chamber. Abnormal phenomena are observed.

## Results and Discussions

**Figure 1:**
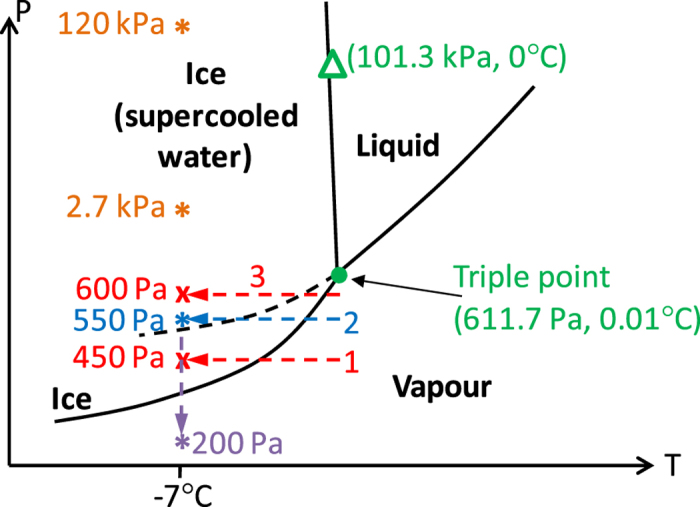
A schematic phase diagram and the phase transition experimental paths of water (not to scale). Paths 1–3 (along with the horizontal arrows) and the pointing down arrow show the water phase transition experimental paths. The 2.7 kPa and 120 kPa points are the estimated inner pressures of the water droplets, which are addressed later in the text.

The dynamic gas-liquid-solid phase transition processes of water have been tracked by SEM with an imaging rate of 50 frame/min. A schematic phase diagram and the water phase transition experimental paths are shown in Fig. 1. To better display the experimental condition points, the diagram is not plotted to scale.

**Figure 2:**
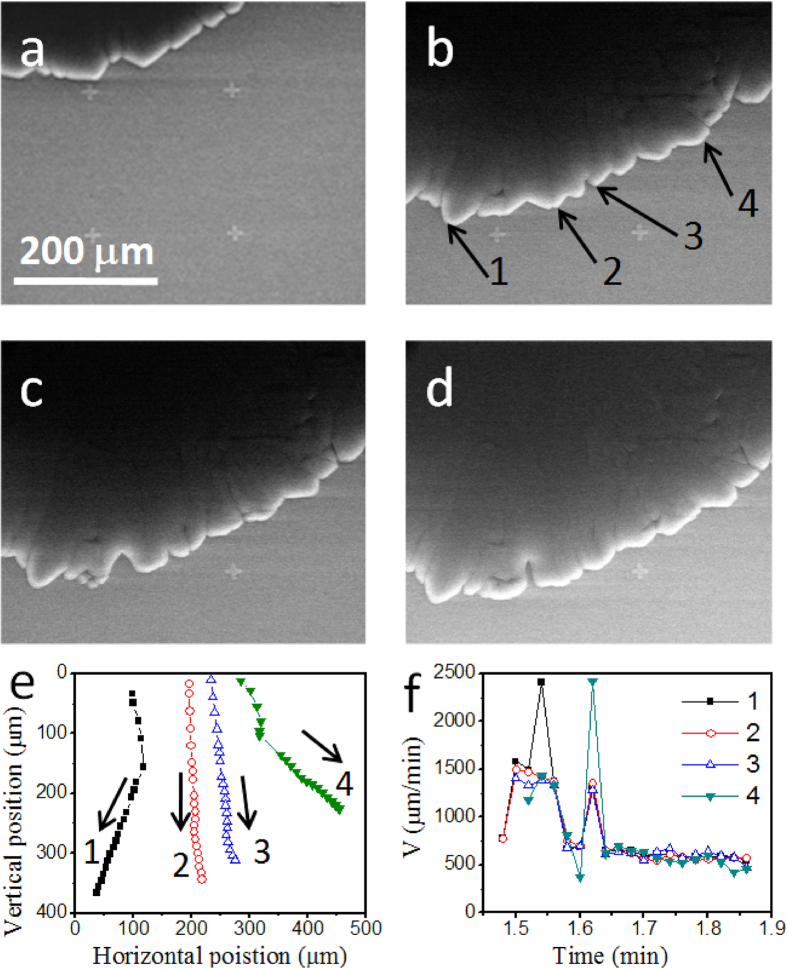
Ice growing process at −7 °C, 450 Pa pressure: (**a**) 1.52 min; (**b**) 1.76 min; (**c**) 1.82 min; (**d**) 1.86 min; (**e**) growth trajectories and (**f**) speeds of several growth fronts as labeled out in (**b**).

Figure 2 shows the data obtained on a SiO_2_/Si substrate at −7 °C and 450 Pa water vapour pressure, following path 1 shown in Fig. 1. Ice crystals grow quickly along the substrate surface through desublimation, without forming any liquid droplets. The ice front is brighter than the nearby regions due to the higher secondary electron escaping possibility and the resulted higher signal level at the edge locations. To better demonstrate the growth trend on the substrate, metal (Cr/Au) marks (the cross-shaped marks shown in Fig. 2) are patterned on the sample surface by photolithography. The ice layer is flat and attaches to the substrate surface, indicating the growth speed along the substrate surface is much higher than that along the surface normal. Solid ice is a thermal insulating material, which has long been used to build igloos by the Eskimos. It can be expected that the heat released during desublimation builds up on the ice surface, resulting in an increased temperature on the ice surface comparing with the exposed substrate, and retards the further ice growth perpendicular to the substrate. The irregular shape of the ice suggests it is not a single crystal (which normally has a regular shape)^16^.

When the ice growth front passes a mark on the substrate, a dent is formed and then left behind (Fig. 2c,d), confirming the ice growth is mainly resulted from new water molecule condensation onto the growth front, instead of a forward sliding motion of the whole ice plate. Several growth fronts are marked out, with their growth trajectory and speed plotted out in Fig. 2e,f, respectively. It can be seen that although the growth directions vary from point to point, the growth speeds showed a similar trend, all stabilized to ~500–600 μm/min. When the growth front meets a cross mark, besides forming a dent, the local growth speed increases suddenly. The two speed peaks of ~2500 μm/min are resulted from such accelerated growth events, probably induced by the different surface properties of the marks from the SiO_2_ substrate.

Besides the two high peaks, it is surprising to see there is a coincident speed oscillation at the beginning for all the four growth fronts, with the high level being ~1500 μm/min and the low level being ~600 μm/min. The desublimation procedure releases heat, so that a fast growth of ice may cause a temperature increase of the local substrate surface, and trigger a slow growth mode; on the other hand, the slow growth mode may allow the temperature to decrease, and eventually trigger a fast growth mode. When the whole body of the ice grows larger, the total heat release during desublimation may be stabilized, so that the temperature and growth speed are stabilized.

**Figure 3:**
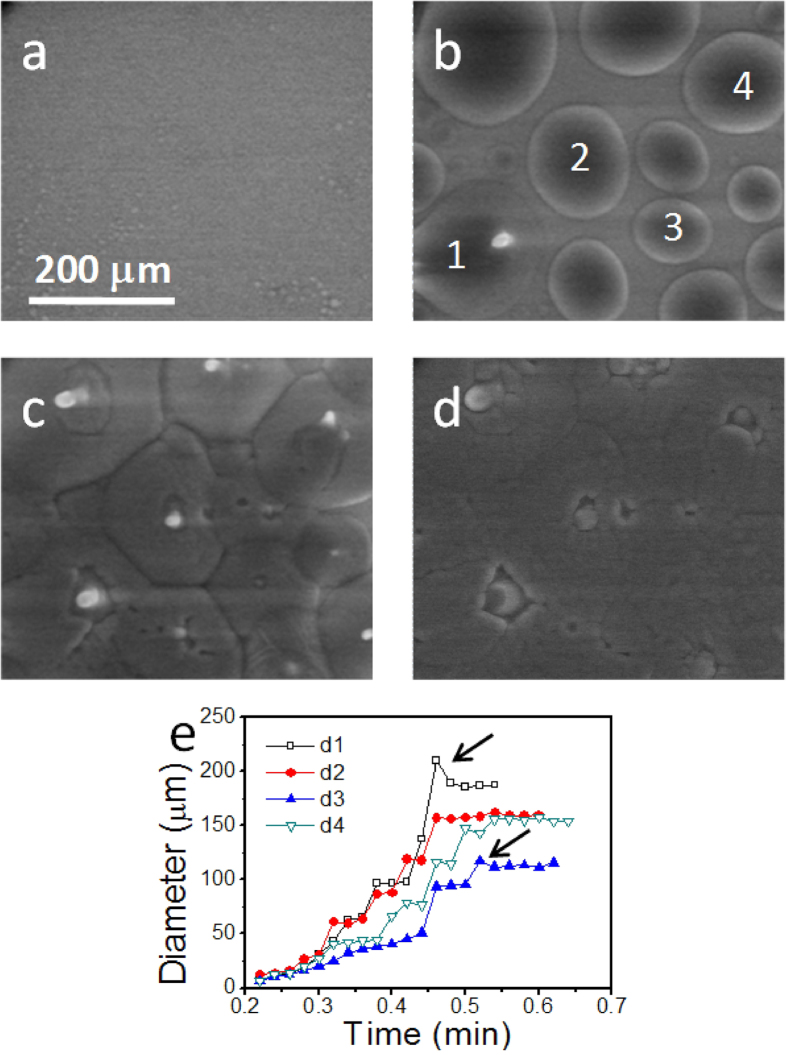
Water phase transition behaviour observed at −7 °C, 550 Pa pressure: (**a**) 0.20 min, tiny droplets formed; (**b**) 0.56 min, when an ice nucleus just forms; (**c**) 0.72 min, when the full viewing region is solidified; (**d**) 1.00 min; (**e**) size vs. time plot for the four typical droplets labeled out in (**b**). The droplet sizes are tracked until ice nuclei are observed in them.

The condensation process is further observed at −7 °C temperature and 550 Pa water vapour pressure, following path 2 shown in Fig. 1. The substrate is smooth and bare in the beginning. Figure 3a shows, as the environmental pressure is increased, water droplets condensate on the substrate surface first, with the sizes being as small as 2.5 μm (limited by the image resolution under our experimental condition). Then the droplets grow/agglomerate into larger ones with time. The newly merged droplets relax into roundish shapes quickly, confirming they are liquid droplets. For the majority of the merging events, the larger droplets eat up the smaller nearby droplets. It should be noted that under 550 Pa, there is no stable liquid water phase according to the phase diagram, which will be addressed again later. Several typical water droplets are labeled out in Fig. 3b, and their sizes are tracked from the early stage until ice nuclei appear in them (Fig. 3e). The growth curves generally consist of slow growth slopes and sudden increase steps, in which the slow slopes are resulted from gradual growth of single droplets, and the sudden increases are caused by droplet-merging events. Besides the general growth trends, after a merging event, the newly merged droplet will relax from an elongated shape to a roundish shape with time, resulting in a lateral size reduction, as pointed out in Fig. 3e. The shape relaxation processes are generally slower for the larger sized droplets, and thus are more easily seen in the large sized droplets. After a quick size reduction, the shape relaxation can continue for some time, which competes with the size increase trend, and results in a flattened growth curve.

When liquid droplets grow to ~100–200 μm sizes, ice crystals start to nucleate inside them (see the white dot in droplet 1 Fig. 3b). The ice nuclei grow up and the water droplets get fully solidified at the time of 0.72 min (Fig. 3c). As the time passes, the ice continues to grow, and the boundaries between ice domains get blurred in the image (Fig. 3d). The roughened granular surfaces as seen in Fig. 3c suggest even the ice formed in a single liquid drop is not a single crystal. As shown in Fig. 3e, the nucleation events in the droplets tend to happen only when their sizes become as large as about 100–200 μm. More quantitative analyses will be given later.

**Figure 4:**
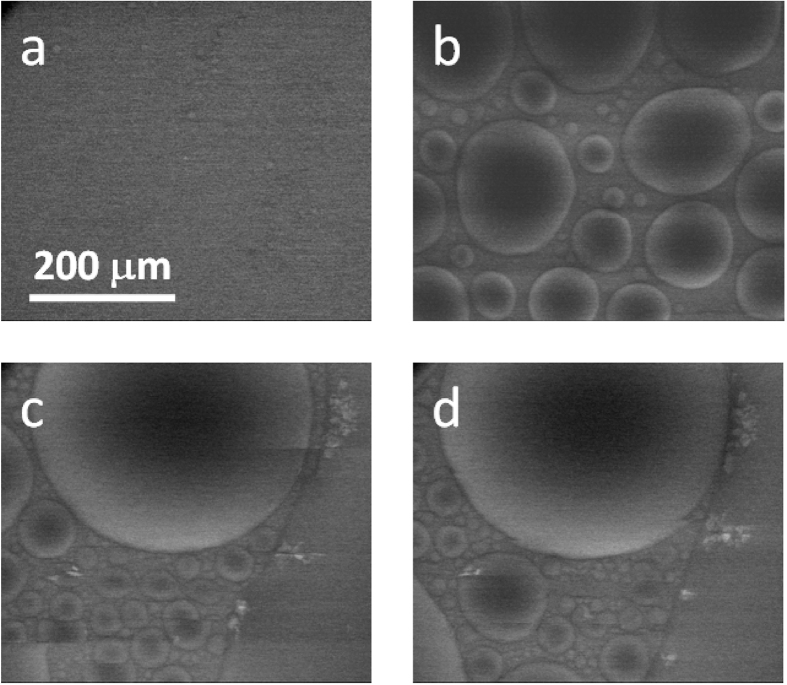
Water phase transition behaviour observed at −7 °C, 600 Pa pressure: (**a**) 0.18 min; (**b**) 0.50 min; (**c**) 1.84 min; (**d**) 1.86 min. (**c**) and (**d**) are taken from a nearby location.

Following path 3 shown in Fig. 1, at −7 °C temperature and 600 Pa water vapour pressure, water droplets first condensate on the substrate surface (Fig. 4a), and then grow up with time (Fig. 4b). Similar to the process in Fig. 3, the liquid droplets grow up and merge together with time. The larger droplets eat up the smaller nearby ones in most of the merging events. And right after a merging event, the droplet relaxes into a roundish shape quickly. On the other hand, now under 600 Pa, the water droplets continue to grow, and are harder to get solidified. As the growing water droplets soon covers the full viewing region, Fig. 4c,d are taken from a nearby area. Some tiny ice crystals can be seen floating in the large water drops, and are hardly grow up with time (Fig. 4c,d). Comparing with the lower pressure cases, liquid water exists for a much longer time under 600 Pa before it finally changes into ice. Note that each of the droplet in Figs 3 and 4 shows a dark outline surrounding a bright edge area, and when a droplet is not very small, the center part of the droplet is dark and gradually become brighter toward the edge. Such kind of image contrasts further confirm the droplets are liquid and round shaped, in contrast to the thin layer shape of the ice shown in Fig. 2.

**Figure 5:**
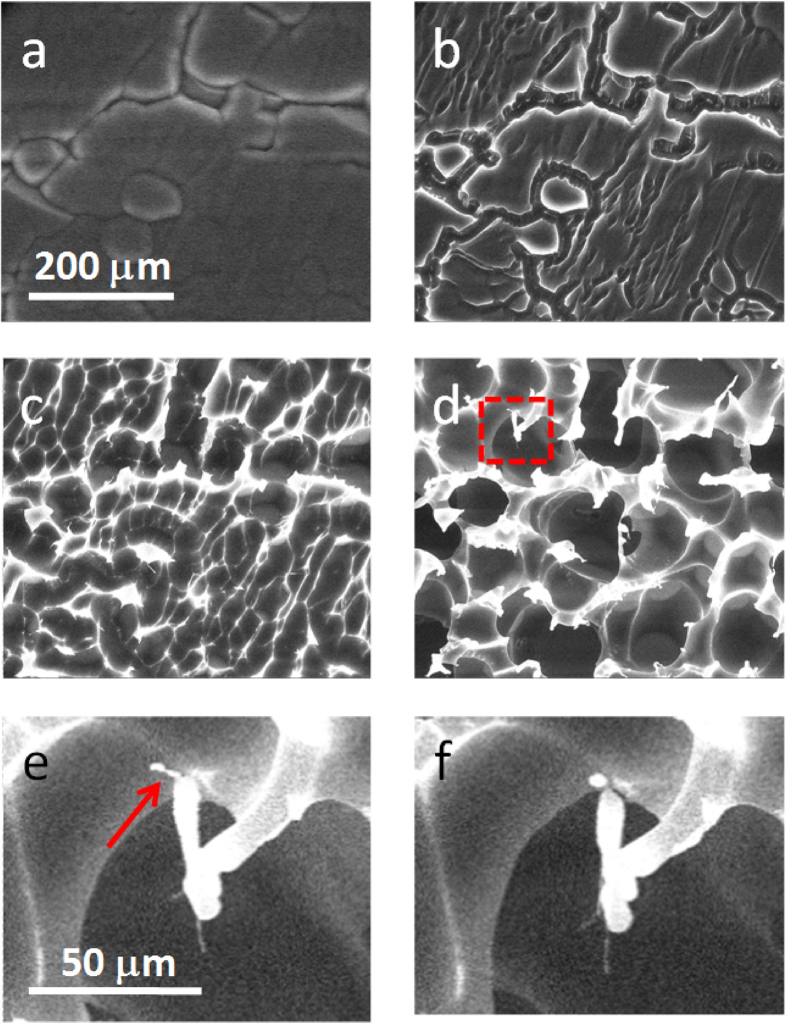
Ice sublimation behaviour observed at −7 °C, 200 Pa pressure: (**a**) 0.02 min; (**b**) 0.40 min; (**c**) 0.80 min; (**d**) 2.00 min. (**e**,**f**) magnification of the framed region in (**d**) taken at 2.00, and 2.08 min, the arrow pointed micro thread section changed to a droplet shape with time. (**a**–**d**) Have the same magnification, and (**e**,**f**) have the same magnification.

Ice sublimation experiments are performed by decreasing the environmental pressure at a fixed temperature. Figure 5 shows a typical set of data obtained at −7 °C after water vapour pressure is decreased to 200 Pa. The ice sample formed originally at −7 °C, ~500 Pa, with its surface fully covered by granular ice (Fig. 5a). Besides the deep micro groves, dim line structures are also seen in the image, separating the larger grains into smaller subgrains. Soon after the pressure is lowered, the groves are observed to become deeper and wider, indicating a higher local sublimation speed. The dim line structures inside the large grains also gradually change into clear trenches (Fig. 5b). Later, the ice surface gets even rougher and turns into a porous structure (Fig. 5c). The pores expand and fuse together with time, forming wall structures in between. Then holes appear and grow up in the walls. As the holes grow up, the walls become disconnected, with micro ice threads left behind (Fig. 5d). Although the process is dominated with sublimation, the micro threads are observed to change their shape with time and some of them even become droplet-like shaped, showing a liquid-like nature (Fig. 5e,f). The ultrafine threads seen in the center of the lower part of Fig. 5e,f keeps the linear shape for a pretty long time.

Our experiments are done below the commonly known gas-liquid-solid triple point pressure of water (611.657 Pa). Thus when the temperature is lowered, one would expect a direct transition from the vapour phase into the ice phase, without forming liquid water (Path 1 shown in Fig. 1), which is consistent with the results shown in Fig. 2.

Note the phase diagram illustrates the conditions of macroscopic materials in the equilibrium states, which might be different from the dynamic/non-equilibrium states in the micro world. It is well known that a metastable liquid state of water may exist below the freezing temperature^2^,^3^,^4^, which is called supercooled water. As illustrated in Fig. 1, in the region above the dashed curve (metastable phase boundary of supercooled water, MPB), ice and supercooled water may actually co-exist under nonequilibrium conditions. When the pressure is close enough to the triple point pressure, the phase transition may follow Path 2 in Fig. 1. After the water material reaches the region where both ice and supercooled water may co-exist, it becomes possible for the vapour to condense into supercooled water. As the supercooled water is metastable, it may further get solidified into the stable ice phase. Such a scenario is consistent with the results shown in Fig. 3. Although Path 2 passes through the exclusive ice region, when the temperature change is quick enough, it is possible that no phase transition happens when passing that region.

How water and ice nucleate on a surface is an important question^21^. Early DFT analysis has shown that as the nucleation and growth of ice need to connect several hydrogen bonds to the substrate or ice surface, the growth of ice layer is pretty unfavorable; and due to the disordered structures of liquid water, water droplets are more favorable to nucleate on a substrate surface^22^. This is in agreement with Fig. 3, where, instead of direct ice nucleation, liquid water droplets first appear on the substrate, and then ice crystals nucleate in the water droplets. Interestingly, in our observation, the supercooled water is not obtained from cooling down a regular liquid water, but forms from the condensation of the water vapour.

Young-Laplace equation describes the relationship between surface tension σ and the resulted pressure change Δ*p* in a liquid (including the supercooled water), which gives the following:^23^


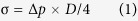


where *D* equals to the spherical diameter of the curved water surface. Thus Δ*p* = 4σ/*D*. Note the roundish shape and movable nature of the droplets confirm they are liquid. The darker contrast in the center part and the gradually increased brightness toward the edge indicate the top of the droplets have spherical surfaces. Therefore Young-Laplace equation is applicable. At −7 °C, the surface tension of supercooled water is about 77 mN/m^23^, which is 77 kPa·μm. The *D* value of a water droplet can be roughly estimated from the SEM image. By adding up with the environmental pressure (550 Pa), the total inner pressure *P*_tot_ can be further estimated. The smallest water droplets observed in our experiments are of ~2.5 μm diameters. The estimated *P*_tot_ of a 2.5 μm sized droplet is ~120 kPa. As shown in Fig. 1, even under such high inner pressure, the droplets still keep the supercooled water nature. From Fig. 3 and the related data, we observe that when ice starts to nucleate in the droplets, the diameter of the droplets is in the range of 100–200 μm. Table 1 lists the actual droplets’ diameters (obtained in Fig. 3e) and the corresponding calculated inner pressures right before ice nuclei form in them.

For the transition from supercooled water into ice, one need to consider the MPB in Fig. 1. Below the MPB, supercooled water is forbidden, and vapour is expected to desublimate directly into solid ice, as observed in Fig. 2. Above that MPB, supercooled water is metastable and has the tendency to transform into the more stable ice phase. The higher pressure, the less tendency to transform into ice, and vice versa. Due to the higher inner pressure in the smaller sized droplets, ice nuclei cannot form in them. Our experimental results (Fig. 3 and Table 1) show, when the inner pressure reduced to ≤2.7 ± 0.5 kPa, ice nuclei can form in the suppercooled water droplets. The observed pressure at which ice nucleates in a droplet is within a range. This is because, besides the pressure, there are other factors that can also affect the ice nucleation and growth, such as impurities and substrate conditions, etc.

When the environmental pressure is further increased, approaching the triple point (Path 3 in Fig. 1), the formation of supercooled water is expected to become easier, and the nucleation/growth of ice crystals is expected to become much harder. Thus liquid water may exist for a longer time without fully transforming into ice, as shown in Fig. 4.

**Figure 6:**
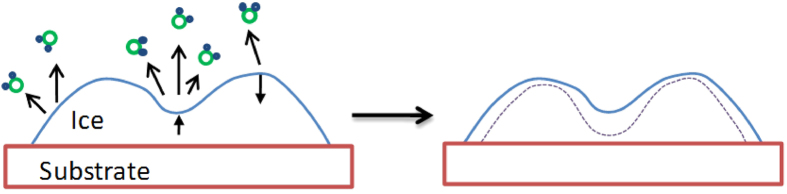
A schematic model for ice morphology evolution during sublimation process. Due to the higher local sublimation speed in the concave regions, dents become deeper and the surface becomes rougher with time (the dashed line represents the later shape of the interface).

For the sublimation process, Fig. 5b shows grain boundary enhances sublimation. However, other defective locations such as protrusions are more stable than their surrounding regions. Surface roughness increases during sublimation have been reported on ice crystal facets with no grain boundary^16^,^24^. Therefore, a defect mechanism cannot fully explain the surface roughness and porous ice structure formation behaviour. Here we propose a model as shown in Fig. 6. Ice-vapour phase transition is sensitive to the pressure and stress. At a fixed temperature, a lower pressure environment shall increase the ice sublimation speed, and a higher pressure environment shall reduce the sublimation speed. When a roughness exists on an ice surface, due to surface tension effect^25^, stress would build up in the ice material correlated to the surface curvatures. At the protruded regions, the stress shall be compressive, and molecules just under the protruded surface will be under a higher pressure and have lower chances to transform into a vapour state, resulting in a lower sublimation speed. While at the concave regions, the stress shall be tensile, with an effect similar to the pressure decrease inside the liquid, which will increase the sublimation speed. In such a model, the decisive events are the sublimation events initiated under the surface: hydrogen bonds are more easily broken in the lower inner pressure regions, generating free water molecules. These molecules will subsequently diffuse out and get released into the vacuum chamber. Such Young-Laplace effect on the sublimation-induced solid morphology change is the opposite of the Young-Laplace effect on the liquid morphology change, where liquid in protruded high inner pressure regions flow to flat/concave regions, and the droplet surface tend to become smoother with time.

With this model, the observed phenomenon in Fig. 5 can be explained as follows: in the beginning, molecules at the concave regions of grain boundaries sublimate faster, and the ice surface roughness increases; later, pores form, and the pores sizes increase with time. As shown in Fig. 5e,f, the surfaces of the pores are smooth, with the defects such as the grain boundaries being hardly visible, supporting the surface morphology instead of the crystalline defect is the major factor determining the porous morphology formation. This sublimation rate-induced morphology change model is different from the expectation from a Gibbs-Thomson effect^26^. Here, near the solid-vapour phase boundary of the phase diagram, the pressure/stress related sublimation dominates and controls the morphology evolution.

A further experiment is performed, and shows that the ice sublimation speed strongly correlates with the environmental pressure, with the sublimation speed being much higher at lower pressure than that at higher pressure situation (see supporting material Fig. S1). These results support our above model, and confirm the porous structure formation during sublimation is repeatable. Note this scenario is opposite to the intuitive understanding, that without considering the pressure/stress in the ice, molecules evaporate in a concave region would have a larger chance to collide with the local surface and get re-deposited, which would reduce the local sublimation speed.

The formation of liquid like droplets during the sublimation process as seen in Fig. 5e,f has some significance. According to the phase diagram shown in Fig. 1, below the triple point pressure, liquid water can only exist in a metastable state, while ice is the stable phase. Thus the transition from ice to liquid water should be forbidden under that environmental pressure^4^. However, due to surface tension effect, the inner pressure/stress should increase in the micro sized material seen in Fig. 5e,f, and help stabilizing a liquid phase, which effect is commonly applied in ice skating. Estimated with the Young-Laplace equation, the inner pressure/stress increases in a micro sized water/ice droplet is quite noticeable (the inner pressure increase of a droplet with diameter of 2 μm is 150 kPa), but due to the very steep condensation line of water (as shown in Fig. 1), such a high pressure is still not enough to liquefy bulk ice at our temperature setting of −7 °C. Thin water layers called quasiliquid layers (QLLs) are known to exist on ice surface even below the melting temperature, which is linked to the phenomena such as ice surface lubrication and morphological changes of snow crystals^25^,^27^. Such QLLs may contribute to the present liquid-like droplets formation. Using confocal optical microscopy, liquid-like QLLs have recently been observed to emerge from ice crystal surfaces^28^. For the ultrafine threads, the high stress inside it might greatly reduce the sublimation speed, so that it can exist for a relatively long time without getting sublimated away, which remains to be further studied.

## Conclusions

*In situ* ESEM observations of water gas-liquid-solid phase transitions are realized for the first time. The experiments are performed below the triple point pressure of water. By cooling down the sample to −7 °C at a low pressure of 450 Pa, water vapour is observed to desublimate directly into ice, with the ice growth speed along the substrate surface being much higher than that along the substrate normal. The growth speed along the substrate surface varies from 1500 μm/min to ~600 μm/min with time, with sudden speed jumps to ~2500 μm/min when the growth front meets a metal mark on the surface.

At a relatively high pressure of 550 Pa, although ice is the stable phase at low temperature (−7 °C) in the phase diagram, water vapour firstly condensates into supercooled water, which transforms into ice later through nucleation and growth processes. The ice nucleation events are observed exclusively in the droplets larger than 100–200 μm sizes. After the droplets are fully solidified, the ice continues to grow through desublimation process. When cooling the sample at an even higher pressure of 600 Pa, the supercooled water becomes relatively stable. It remains in the liquid state for a long time, with only a few ice nuclei floating in it.

At −7 °C, when the pressure is lowered to 50–200 Pa, ice is observed to sublimate into vapour phase directly. During the sublimation, the ice surface gets rougher with time, and porous structures eventually form. Although it is forbidden to form an equilibrium liquid phase in the low pressure sublimation process, liquid like droplets are observed during the procedure.

Surface tension and the resulted inner pressure change of water are shown to have important effects to the micro water phase transitions. ESEM is demonstrated to be useful for studying water three phase transitions and the present results deepen the understanding of the water phase diagram.

## Methods

**Figure 7:**
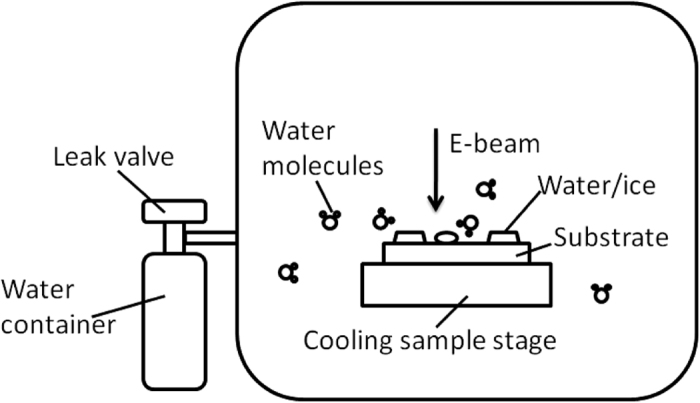
Schematic of the experimental setup.

ESEM, FEI Quanta 600 FEG, is used for the *in situ* water phase transition experiments. The background pressure of the system is about 10^−3^ Pa. Water vapour is introduced into the chamber through a leak valve. A Deben MK3 Cool-stage is used as the cooling stage to adjust the substrate temperature, which works between −30 °C and the ambient temperature. Secondary electron images are taken with a 30 keV electron beam. A SiO_2_ (300 nm) coated Si wafer is used as the substrate. Cr/Au marks (5 nm/70 nm thicknesses) have been patterned on the substrate using electron beam deposition and photolithography. For the condensation experiments, firstly the water vapour pressure is set to a value between 450 and 600 Pa (450, 550, and 600 Pa) when the substrate temperature is 0 °C, and then the sample temperature is decreased to −7 °C. In these experiments, the substrate temperature drop to −5 °C within 10 s after setting the temperature, and then gets stabilized to −7 °C in about 1 min. 0 min is defined as the moment when the temperature setting is just switched to −7 °C. For the evaporation experiments, the sample temperature is first stabilized at −7 °C at a relatively high pressure, then the vapour pressure is decreased to a value between 50 to 200 Pa (In the supplementary material, we further display an experiment, where besides the pressure changes, the temperature setting is increased to 2 °C at the same time). To better track the phase transition processes, images are automatically taken with a speed setting of 50 frame/min after an experiment is started. Without further mention, each set of the presented SEM images in the figures are of the same magnification, taken at the same location. A schematic of the experimental set up is shown in Fig. 7.

## Additional Information

**How to cite this article:** Chen, X. *et al*. Abnormal gas-liquid-solid phase transition behaviour of water observed with *in situ* environmental SEM. *Sci. Rep.* **7**, 46680; doi: 10.1038/srep46680 (2017).

**Publisher's note:** Springer Nature remains neutral with regard to jurisdictional claims in published maps and institutional affiliations.

## Supplementary Material

Supplementary Materials

## Figures and Tables

**Table 1:** Droplets’ diameters and the calculated inner pressure changes.

Droplet number	D (μm)	ΔP (kPa)	P_tot_ (kPa)
1	188	1.6	2.2
2	160	1.9	2.5
3	116	2.7	3.2
4	154	2.0	2.5
