# Supplementary material for: Abnormal gas-liquid-solid phase transition behaviour of water observed with in situ environmental SEM
Source: Sci Rep. 2017 Apr 24;7:46680. doi: 10.1038/srep46680 (PMC5402395; doi:10.1038/srep46680)
Supplement: Supplementary Materials [file srep46680-s1.pdf]

Supplementary material:
Abnormal gas-liquid-solid phase transition behaviour of water observed with *in situ* environmental SEM

Xin Chen, Jiapei Shu, & Qing Chen

Ice sublimation experiments are performed at different pressures and temperatures. Start with a temperature setting of -7°C and a water vapour pressure around 500 Pa, after the substrate is fully covered with ice, the pressure is lowered to check the ice sublimation behavior. Fig. S1a-c show images taken after the pressure is lowered for 0.4 min. In Fig. S1a, with the pressure lowered to 200 Pa, it is seen the grain boundary regions sublimate quickly, leaving trenches on the sample. The inner grain region surfaces showed increased roughness but are still largely connected. In Fig. S1b, with a pressure of 100 Pa, after 0.4 min sublimation time, not only the grain boundary regions become trenches, the inner grain regions have also sublimated extensively, and become very porous. Fig. S1c shows, with a pressure of 50 Pa, after 0.4 min time, the sublimation has preceded to a further stage, in which the original grain boundaries and inner grains have become indiscernible, and the pore walls have become very thin. This data demonstrated that the lower the environmental pressure, the stronger ice sublimation effect.

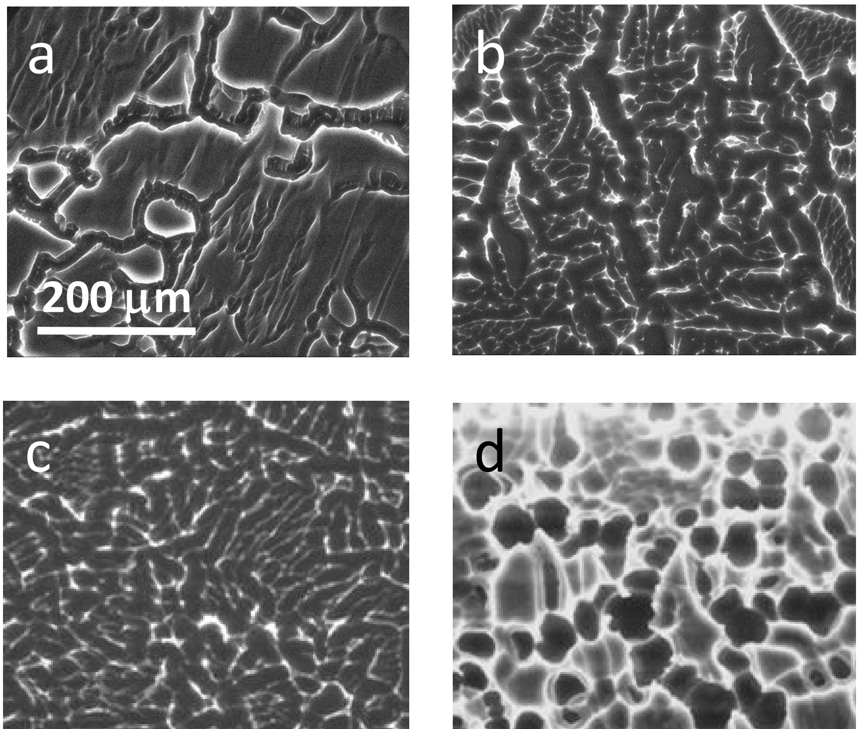

Figure S1 Ice sublimation behaviour observed at 0.4 min, at different pressure and temperature: (a) -7°C, 200 Pa; (b) -7°C, 100 Pa; (c) -7°C, 50 Pa; (d) 2°C, 200 Pa. The four images have the same magnification.

Comparing Fig. S1a-c with Fig. 5a-d, it can be also seen that after the ice surfaces become fully covered with porous structures, the obtained pore wall thicknesses (and characteristic feature sizes) are smaller for a lower water vapour pressure.

In a further experiment, with temperature stabilized at -7°C, after the substrate is fully covered with ice, not only the pressure is lowered to 200 Pa, the temperature setting is also increased to 2°C at the same time (Fig. S1d). Comparing with Fig. S1a, it can been seen after at the same sublimation time of 0.4 min, the ice surface is now fully covered with porous structures, and the original grain boundaries and inner grains become indiscernible. This result indicates the sublimation effect is stronger at the higher temperature, and altered the ice surface more extensively within a certain amount of time.

Comparing Fig. S1d with Fig. 5c, it can been further seen that under the same environmental pressure, after the ice surfaces become fully covered with porous structures, the obtained pore wall thicknesses (and characteristic feature sizes) are larger with a higher substrate temperature.
